# *Dicoma anomala* Enhances Phthalocyanine Mediated Photodynamic Therapy in MCF-7 Breast Cancer Cells

**DOI:** 10.3389/fphar.2022.892490

**Published:** 2022-04-26

**Authors:** Alexander Chota, Blassan P. George, Heidi Abrahamse

**Affiliations:** Laser Research Centre, Faculty of Health Sciences, University of Johannesburg, Johannesburg, South Africa

**Keywords:** *Dicoma anomala*, breast cancer, photodynamic therapy, ZnPcS_4_, MCF cell lines

## Abstract

Breast cancer is one of the most common types of cancer in women, and it is regarded as the second leading cause of cancer-related deaths worldwide. The present study investigated phytochemical profiling, *in vitro* anticancer effects of *Dicoma anomala* methanol root extract and its enhancing effects in phthalocyanine mediated PDT on MCF-7 (ATCC^®^ HTB-22™) breast cancer cells. Ultra-high performance liquid chromatography coupled to electrospray ionization quadrupole-time of flight mass spectrometry (UHPLC-qTOF-MS^2^) was used to identify the secondary metabolites in the crude extract. The 50% inhibitory concentration (IC_50_) of the two experimental models was established from dose response studies 24 h post-treatment with *D. anomala* methanol root extract (25, 50, and 100 μg/ml) and ZnPcS_4_ (5, 10, 20, 40, and 60 μM) mediated PDT. The inverted microscope was used to analyze morphological changes, trypan blue exclusion assay for viability, and Annexin V-fluorescein isothiocyanate (FITC)-propidium iodide (PI) for cell death mechanisms. Immunofluorescence analysis was used to investigate the qualitative expression of the Bax, p53, and caspase 3 apoptotic proteins. Experiments were performed 4 times (*n = 4*) and SPSS version 27 software was used to analyze statistical significances. *D. anomala* methanol root extract induced cell death in MCF-7 cells by decreasing cell viability. The combination of *D. anomala* methanol root extract and ZnPcS_4_ mediated PDT led to a significant increase in apoptotic activities, expression of Bax, and p53 with significant decrease in cell viability. These findings pinpoint the possibility of *D. anomala* methanol root extract of being employed as a natural antiproliferative agent in the treatment of various cancers.

## 1 Introduction

Cancer is a condition characterized by unregulated proliferation of cells (Wang et al., 2013). The tumor cells may arise from any parts of the body and may develop metastatic abilities which allow them to spread from the site of origin to distant parts of the body (Obenauf and Massagué, 2015). If left untreated, cancer may lead to other serious medical complications and eventually death. Breast cancer is one of the most prominent forms of cancer that mainly affects women (Wang et al., 2021). According to the International Agency for Research on Cancer’s GLOBOCAN projections for 2020, global breast cancer incidence rate was reported to be 2.26 million cases in 2020, with projections of 3.19 million cases by 2040. The mortality rate is also anticipated to rise from 685 thousand of 2020 to 1.04 million by 2040. In Africa, the incidence rate of breast cancer was estimated at 187 thousand in 2020 and estimated to rise to 347 thousand by 2040, while the mortality rate was estimated at 85.8 thousand in 2020 and projected to rise to 162 thousand by the year 2040 (World Health Organization, 2020). Rise in incidence and mortality rates of breast cancer can be ascribed to a number of risk factors which include alcohol consumption, age, hormones, genetic predispositions, and unhealthy lifestyles (Sun et al., 2017). Common symptoms of breast cancer include nipple retraction, skin dimpling, and blood stained discharge, pain and lump in the breast (Divya et al., 2021). Early detection of these symptoms plays a cardinal role in the treatment and management of breast cancer. Physical examinations, radiological imaging, and laboratory investigations are the most common screening techniques employed in early detection of breast cancer (Barba et al., 2021).

Common conventional treatment modalities used in the treatment of cancer may be curative or palliative. Curative treatments are basically a form of treatment aimed at complete eradication of a disease or an illness. In most cases, curative treatments are either administered as a primary treatment or as an adjuvant. On the other hand, palliative treatments are non-curative forms of cancer treatments. This form of cancer treatment is simply aimed at controlling the progression of cancer from one stage to another (Neugut and Prigerson, 2017). However, the most common treatment modalities used in the treatment of breast cancer include surgery, radiotherapy, chemotherapy, and hormonal therapy. In addition, many cancer patients may receive surgery and radiotherapy as primary/routine therapy and an adjuvant which could either be hormonal or chemotherapy (Whelan and Levine, 2005).

Photodynamic therapy (PDT) is a localized form of cancer therapy that uses high energy beams to excite drugs known as photosensitizers (PS). In an excited state, PS’s interactions with molecular oxygen (O_2_) results in the generation of highly cytotoxic reactive oxygen species (ROS) that may cause tumor cell death via oxidative damage of vital organelles of the cell e.g., the mitochondria, endoplasmic reticulum (ER), and peroxisomes (Lee et al., 2021). Despite it being a promising alternative or supportive therapeutic option, PDT has its own limitations. Some of the common limitations of PDT include lower light penetration in tissues, lack of water solubility, low singlet quantum yield, and lack of specificity of certain PSs (Gunaydin et al., 2021; Park et al., 2021). Zinc phthalocyanine (ZnPc) derivatives, such as zinc phthalocyanine tetrasulfonate (ZnPcS_4_), are second generation PSs that have enhanced tissue penetration, high triplet quantum yields (*Φ*_T_ > 0.4), with longer triplet lifetimes (*τ*_T_ > 100 μs) (Brozek-Pluska et al., 2020). Additional advantages of ZnPcs in cancer PDT include high photo-chemical stability, excitation within the therapeutic window (350–800 nm), and low dark toxicity (Chin et al., 2014; Matlou et al., 2018). Although ZnPcs have been reported to have a better anticancer efficacy in many cancer cell lines, the major drawback is their inherent ability to self-aggregation (Brozek-Pluska et al., 2020). Self-aggregation of phthalocyanines has been reported in many studies for limiting singlet oxygen (^1^O_2_) generation capacity (Chin et al., 2014). Likewise, recent studies have demonstrated that PS’s used in PDT which have some of the highlighted limitations above can be improved through supplementation of a nano-enabled drug delivery system (Park et al., 2021).

The use plant–derived bioactive compound as an alternative therapy in complementary medicine has increased with time. Further, many pharmaceutical companies have shifted their focus from producing synthetic drugs to producing drugs of natural origin (George et al., 2015). The increase in costs, and side effects of conventional therapies has encouraged many people to be more dependent on herbal formulations (James et al., 2018). *Dicoma anomala* belongs to the family Asteraceae, and has shown to provide ethno-medicinal effects in the treatment of a variety of ailments (Tripathy et al., 2020). *D. anomala* is widely distributed in the Sub-Saharan Africa. Literature has shown the presence of different classes of secondary metabolites in *D. anomala* which includes flavonoids, sesquiterpenes, triterpenes, and tannins (Maroyi, 2018). These compounds are reported to possess antibacterial, antiplasmodial, anticancer, anti-inflammatory, and antioxidative properties (Makuwa and Serepa-Dlamini, 2021). Based on the literature, this study was aimed to investigate phytochemical profiling, and dose-dependent anticancer properties of *D. anomala* methanol root extract in monotherapy and in combination with zinc phthalocyanine tetrasulfonic acid (ZnPcS_4_) mediated PDT in MCF-7 breast cancer cells. In addition, the cell death mechanisms by flow cytometry, the expression of apoptotic proteins (Bax, p53, and caspase 3) by immunofluorescence were explored.

## 2 Materials and Methods

### 2.1 Plant Collection, Identification and Extraction

*D. anomala* was collected from the highlands of Zambia’s Eastern province in April 2020 (13.6445°S, 32.6447°E). The plant was authenticated by Zambia Agriculture Research Institute (ZARI) (phytosanitary certificate SR No: 0006064). The root of *D. anomala* was thoroughly washed using running tap water and shade dried. The dried root was powdered using a blender and used for the extraction. About 10 g of fine powder was extracted in Soxhlet using methanol (70%) at 50°C for 36 h. The extract was dried and kept in the dark at room temperature and pressure (rtp) for further investigations.

### 2.2 Phytochemical Profiling of *Dicoma anomala*

#### 2.2.1 Sample Preparation for UHPLC-qTOF-MS^2^

About 10 mg of *D. anomala* root extract (methanol) was dissolved 10 ml of D. H_2_O. Sample was vortexed at rtp for 40 min. A 0.22 μm membrane syringe filter was used to filter the homogeneous extract. For phytochemical profiling, 1 ml of *D. anomala* methanol root extract was injected in the ultra-high performance liquid chromatography coupled to electrospray ionization quadrupole-time of flight mass spectrometry (UHPLC-qTOF-MS^2^) for identification of secondary metabolites.

#### 2.2.2 UHPLC-qTOF-MS^2^ Analytical Procedure

A Waters Synapt G1 HDMS mass spectrometer was used in combination with a Waters Acquity UPLC system for the study. The Waters Acquity T3 HSS C18 column, measuring 150 mm × 2.1 mm × 1.8 m, was employed for the analysis. The mobile phase constituted of 0.1% (v/v) formic acid (CH₂O₂) in H_2_O for solvent A and 0.1% (v/v) CH₂O₂ in acetonitrile (C_2_H_3_N). The separation of solvent A started with 100% of composition and decreased up to 1% in 16 min and then held constant for 1 min before returning to 100% after 1 min giving a re-equilibrium time of 2 min which resulted in 20 min of total run time. The injection volume was 1 µl and the flow rate was 0.4 ml/min. The temperature in the column was kept at 60°C. The detection and analysis of UHPLC-qTOF-MS^2^ were carried out in full MS-SIM (mass spectrometry-selected ion monitoring) conditions, followed by data dependent MS^2^ (dd-MS^2^) with positive and negative polarity switching throughout the scanning window of mass-to-charge ratio (m/z) 50 to 1,200 Da (Da) with a mass accuracy less than 5 parts per million (ppm). For optimal mass spectroscopy analysis conditions, standardized spectrometric reference parameters were used with a capillary voltage of 2.5 kV, a cone voltage of 30 V, and extraction voltage of 4 V, source temperature of 120°C, and desolvation temperature of 450°C. The following conditions were used to set the overall mass resolution for MS: 550 L/Hr for desolvation gas flow (nitrogen), and 50 L/Hr for cone gas flow (nitrogen). The scan took 0.1 s. The data obtained was analyzed using MassLynx v4.1 SCN 872 software.

### 2.3 *In Vitro* Studies

#### 2.3.1 Cell Culture Conditions

Breast cancer cells (MCF-7) (ATCC^®^ HTB 22™) used in this study was obtained from American Type Culture Collection (ATCC). The cells were cultured in T75 culture flask using Dulbecco’s Modified Eagle’s Medium (DMEM), supplemented with 10% Fetal Bovine Serum (FBS), 1% Amphotericin B, 1% Penicillin-streptomycin, and incubated at 37°C, 85% humidity, and 5% CO_2_. Once cells reached 90% confluence, culture flasks containing cells were washed using 10 ml Hank’s balanced salt solution (HBSS) and detached using 3 ml of TrypLE™ Express (TrypLE) (Invitrogen, 12605-028). The cells were seeded at 5 × 10^5^ cell density in 3.4 cm^2^ diameter culture dishes for experiments.

#### 2.3.2 ZnPcS_4_ Photosensitizer Stock Concentration Preparation

The ZnPcS_4_ (sc-264509A) photosensitizer with a molecular weight of 898.15 g/mol was procured from Santa Cruz^®^ Biotechnology and reconstituted to a concentration of 0.0005 M. The stock solution (5 ml) was prepared by dissolving 0.0024 g of ZnPcS_4_ in 5 ml of 0.001 M 1X phosphate-buffered saline (1X PBS). The reconstituted 0.0005 M was wrapped in aluminum foil and kept at rtp in the dark throughout the study. C_1_V_1_ = C_2_V_2_ formula was used for the preparation of the stock solution.

#### 2.3.3 Subcellular Localization of ZnPcS_4_ Photosensitizer

About 3 × 10^5^ cells/mL of MCF-7 cells were seeded onto a 3.4 cm^2^ diameter culture plates with sterile microscopic coverslips. Pre-warmed complete DMEM media (2 ml) was added to the culture plates followed by 1 ml of the cell culture giving a final volume of 3 ml. Cells were then incubated for 4 h and washed three times with 1X PBS and added 3 ml pre-warmed complete media. The cells were then treated with 5 μM of ZnPcS_4_ and incubated for 24 h post-incubation and washing with 1X PBS, cells were fixed with 1 ml of 4% paraformaldehyde and incubated for 15 min at rtp which was followed by washing with 1X PBS for three times. Cells were permeabilized for 15 min at rtp with 1 ml of 0.5% Triton X-100 and then washed thrice with 1X PBS. Washed cells were then incubated at 4°C for 30 min with 200 μl of pre-warmed probes containing 100 nM mitochondrial tracker (Mito-Tracker), 65 nM lysosomal (Lyso-Tracker), and 65 nM endoplasmic reticulum (ER-Tracker), respectively. Cells were washed three times in 1X PBS after incubation, then counter stained for 5 min with 200 μl of 4′,6-diamidino-2-phenylindole (DAPI) and rinsed three times with 1X PBS. The treated cell coverslips were removed from the culture plates, inverted, and mounted onto sterile glass microscopic slides using aqueous mounting solution. The slides were then sealed with nail polish and examined for ZnPcS_4_ organelle localization using Alexa Fluor 594, DAPI, and FITC filters under a Carl Zeiss Axio Z1 microscope.

### 2.4 Dose Response Studies

Cellular dose response studies were performed on MCF-7 cells 24 h post-treatment at various concentrations of *D. anomala* methanol root extract and ZnPcS_4_ mediated PDT using a 680 nm diode laser at a fluency of 10 J/cm^2^ (Table 1). Assays that were performed include morphological analysis, and trypan blue dye exclusion assay for cellular viability. To evaluate the effects of *D. anomala* methanol root extract and ZnPcS_4_ mediated PDT, untreated (control) cells were compared with treatment groups in the two experimental models (Table 2). The following formula was used to determine the irradiation dosimetry for effective PDT: Irradiance (J/cm^2^) = [(power (W)/surface (cm^2^) x Time (s)].

**TABLE 1:** Laser irradiation parameters.

Name	Parameters
Laser type	Semiconductor (Diode)
Wavelength	680 nm
Emission	Continuous Wave (CW)
Spectrum	Red (Visible Light)
Power output	193 mW
Fluency	10 J/cm^2^
Exposure time	8 min 6 s

**TABLE 2:** Experimental models used in dose response studies.

Experimental model	Experimental groups	Description
1	I	Cells only (control)
	*D. anomala*	Concentration (μg/ml)
	II	25
	III	50
	IV	100
II	I	Cells only/cells + 680 nm laser (control)
	ZnPcS_4_	Dark toxicity 680 nm laser (10 J/cm2)
	II	Cells + 5 μM Cells +5 μM + laser
	III	Cells + 10 μM Cells + 10 μM + laser
	IV	Cells + 20 μM Cells + 20 μM + laser
	V	Cells + 40 μM Cells + 40 μM + laser
	VI	Cells + 60 μM Cells + 60 μM + laser

#### 2.4.1 Morphological Analysis

The morphology of untreated and treated cells in each experimental models were analysed by comparing the cellular architecture of cells within images captured by a Wirsan Olympus CKX 41 inverted light microscope attached with digital camera (Olympus C5060-ADUS). This was followed by trypsinization cells using 300 μl of TrypLE and later resuspension of cells in HBSS for further cell viability analysis.

#### 2.4.2 Trypan Blue Exclusion Assay (Cell Viability)

The trypan blue dye (Sigma-Aldrich T8154) exclusion assay was performed to determine the percentage of viable cells in cell suspensions of untreated and treated groups. The principle behind this assay stems from the ability of viable cells with undamaged cellular membrane selectively resisting trypan blue staining. On the other hand, non-viable cells with damaged cellular membranes are unable to resist the stain. In order to quantify the percentage of viable and non-viable cells, equal amount (10 μl) cell suspensions and 0.4% trypan blue dye (1:9) were thoroughly mixed and pipetted (10 μl of homogenous mixture) onto each window of the countess slide. Viability was quantitatively read using an automated countess II FL cell counter.

### 2.5 Combination Therapy of *D. anomala* and ZnPcS_4_ Mediated PDT

**FIGURE 1:**
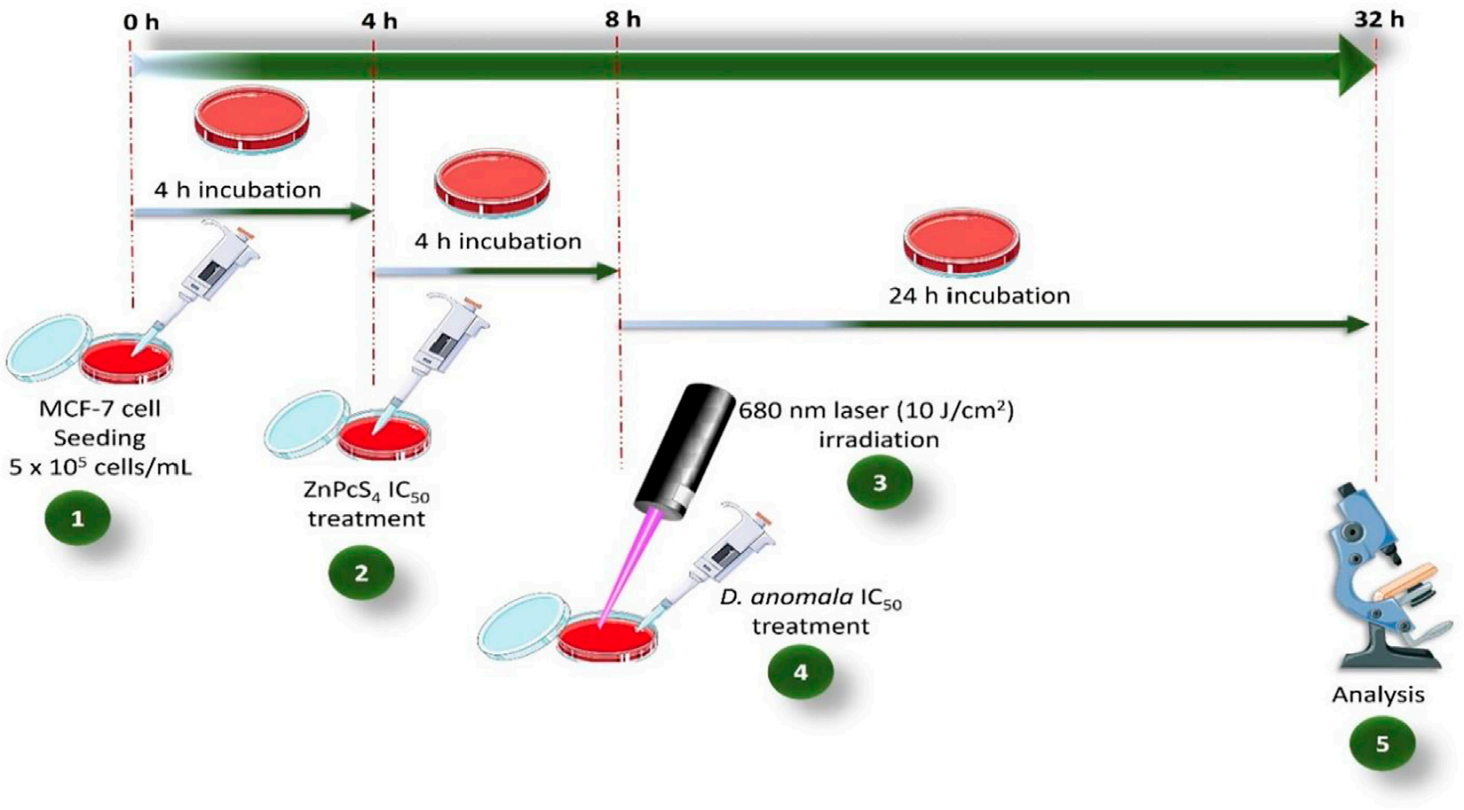
ZnPcS_4_ and *D. anomala* methanol root extract treatment schedule in combination therapy.

The 50% inhibitory concentration (IC_50_) determined from dose response studies for *D. anomala* and ZnPcS_4_ mediated PDT were used in the combination experiments. In combination therapy, *D. anomala* methanol root extract IC_50_ was administered as an adjuvant in ZnPcS_4_ mediated PDT. Following cell culture, the treatment schedule for the combination therapy experiments (*D. anomala* methanol root extract and ZnPcS_4_) is depicted in Figure 1.

#### 2.5.1 Cell Death Analysis

We further evaluated the cell death mechanism using the established IC_50_ from dose response studies of two experimental models. The IC_50_ were administered as individual treatments as well as in combination. After 24 h of treatment, morphology and viability analysis, Annexin V-fluorescein isothiocyanate (FITC)-propidium iodide (PI) flow cytometry, and immunofluorescence (Bax, p53, and caspase 3 expression) experiments were performed.

#### 2.5.2 Flow Cytometry (Annexin V-FITC/PI)

Annexin V-FITC/PI (BD Pharmingen™) (556570) flow cytometry analysis was used to detect the cell death mechanisms in untreated and treated cells. To assess the proportion of cells undergoing apoptosis or necrosis, the Annexin V-FITC-PI kit was utilized according to the manufacturer’s instructions. Briefly, cells were detached from the 3.4 cm^2^ culture dishes by adding 300 μl of TrypLE and incubated for 5 min. Cells were later washed thrice and resuspended in 500 μl of 1X binding buffer. About 100 μl of each cell suspension was dispensed into sterile flow cytometry tubes followed by addition of 5 μl Annexin V-FITC and PI in all experimental groups and incubated for 15 min in dark. After incubation, 400 μl 1X binding buffer was added and incubated for 30 min in the dark. Flow cytometry tubes containing controls and experimental samples were read by using a flow cytometer Becton Dickinson (BD) Accuri™ C6.

#### 2.5.3 Immunofluorescence (Bax, p53, and Caspase 3 Expression)

Immunofluorescence was used to determine the possible cell death pathway induced. Cells were seeded (5 × 10^5^ cells/ml) onto a 3.4 cm^2^ diameter culture plates with sterile microscopic coverslips at the bottom of the culture plate and incubated. Following the 24 h treatment, culture plates containing cover slips with cells were washed three times using 1X PBS. Cells were then fixed for 15 min by adding 1 ml of 4% paraformaldehyde and later washed thrice with wash buffer to remove excess fixative. Cells were permeabilized by adding 1 ml of 0.5% Triton X100 followed by 15 min incubation at rtp. Excess 0.5% Triton X100 was washed off using 1X PBS for three times. To avoid non-specific binding of antibodies, cells were blocked for 1 h at rtp by adding 1 ml of 1% Bovine Serum Albumin (BSA) which was followed by wash with 1X PBS for three times. Primary and secondary antibodies were subsequently reconstituted as per the manufacturer’s instructions and 200 μl of primary antibodies; anti-Bax (WH0000581M1), anti-p53 (SAB5700047), and anti-caspase 3 (C8487) supplied by Sigma were added to the culture plates and incubated for 2 h at rtp in the dark followed by thrice wash using ice cold 1X PBS. Washed cells were then incubated for 1 h at rtp in the dark with 200 μl of secondary antibodies; goat anti-mouse IgG-FITC (sc-2010) and rabbit anti-goat IgG-FITC (sc-2777) supplied by Santa Cruz^®^ Biotechnology. After 1 h of incubation, the cells were rinsed thrice with ice cold 1X PBS and then stained for 5 min with 200 μl of DAPI. The cells were then washed thrice with ice cold 1X PBS before reinverting coverslips onto sterile microscope slides with aqueous mounting media. Thereafter, the slides were sealed with nail polish and analyzed with a Carl Zeiss Axio Z1 microscopic slide scanner. Filters Alexa Fluor 594, DAPI, and FITC were used to look for apoptotic protein expression on the slides.

### 2.6 Statistical Analysis

The statistical analysis of experimental data was performed by using IBM SPSS version 27. All sets of experiments were repeated four times (*n* = 4). One-way analysis of variance (ANOVA) was performed with the aim of determining statistical significance between the control and experimental groups. All experimental values were compared to those of the untreated cancer cells (control). Dunnett test was used with a confidence interval set at 0.95% to analyse the statistical significance between the control and experimental groups. OriginPro 2018 v9.5.1 software was used to plot the bar and scatter graphs. Analyzed data on graphs are represented as mean values, standard errors (±SE) and statistical significance (*p* < 0.05*, *p* < 0.01**, and *p* < 0.001***).

## 3 Results

### 3.1 Phytochemical Profiling of *D. anomala* Methanol Root Extract

**FIGURE 2:**
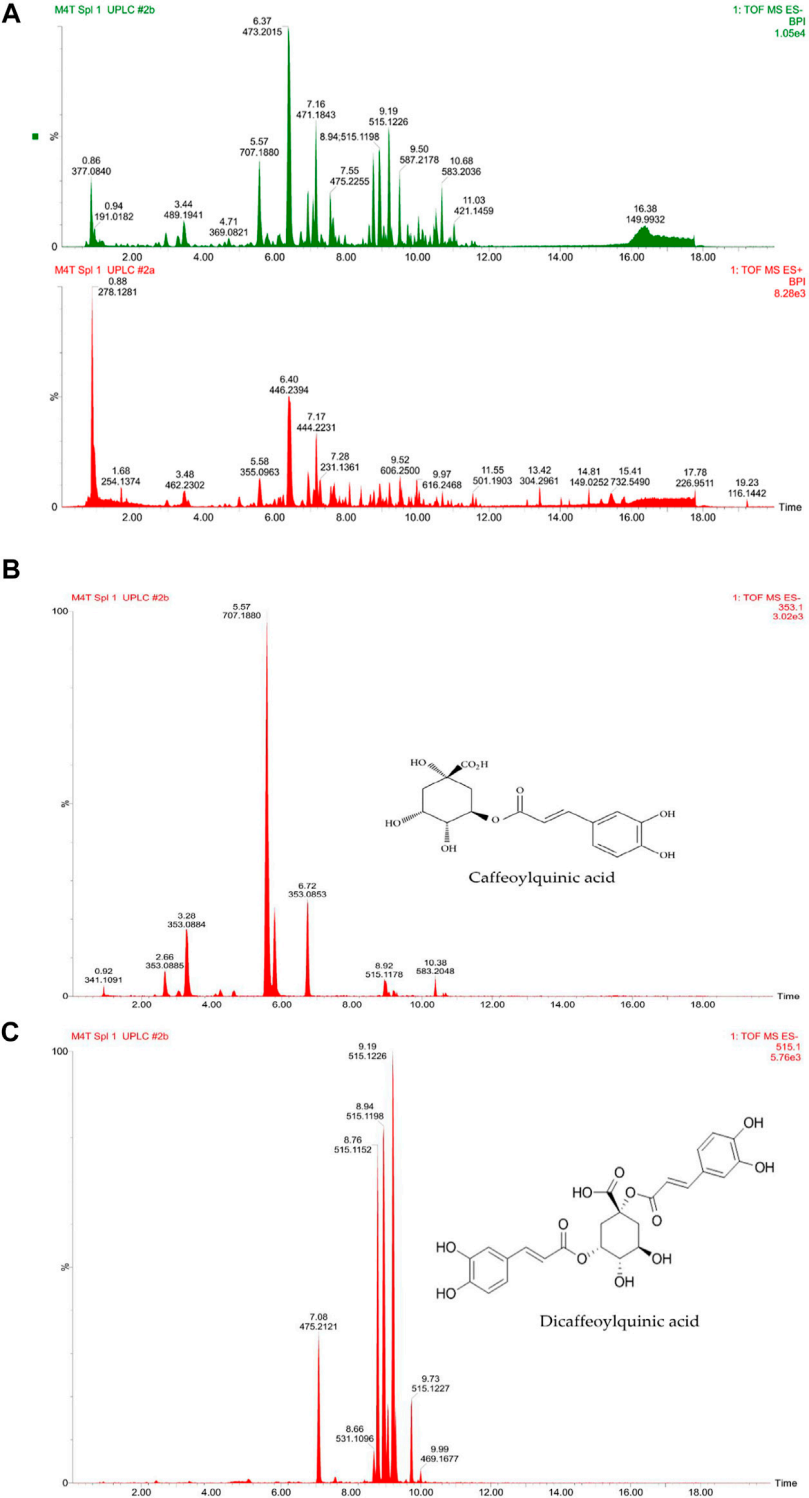
*Dicoma anomala* methanol root extract negative and positive base peak ion (BPI) chromatograms **(A)**, caffeoylquinic **(B)** and dicaffeoylquinic **(C)** acids in negative XIC of *D. anomala* methanol root extract.

The recovery percentage of *D. anomala* methanol root extract was 25%. Figure 2 a shows the total ion UHPLC-qTOF-MS^2^ chromatograms of *D. anomala* methanol root extract in the negative and positive modes, respectively. The presence of phytocompounds in *D. anomala* methanol root extract was as evidenced by multiple major and minor peaks with complete separation by 18 min. The molecular weights, retention time (RT), and peaks of *D. anomala* methanol root extract was used to identify secondary metabolites. Two major secondary metabolites; caffeoylquinic and dicaffeoylquinic acidic compounds were identified in the negative extracted-ion chromatogram (XIC) of *D. anomala* methanol root extract (Figures 2B,C). Fragmentation analysis of UHPLC-qTOF-MS^2^ results was used to identify phytocompounds, which was followed by molecular mass machining with matching molecular masses of known compounds from the mass bank library data base.

### 3.2 Subcellular Localization of ZnPcS_4_ Photosensitizer

**FIGURE 3:**
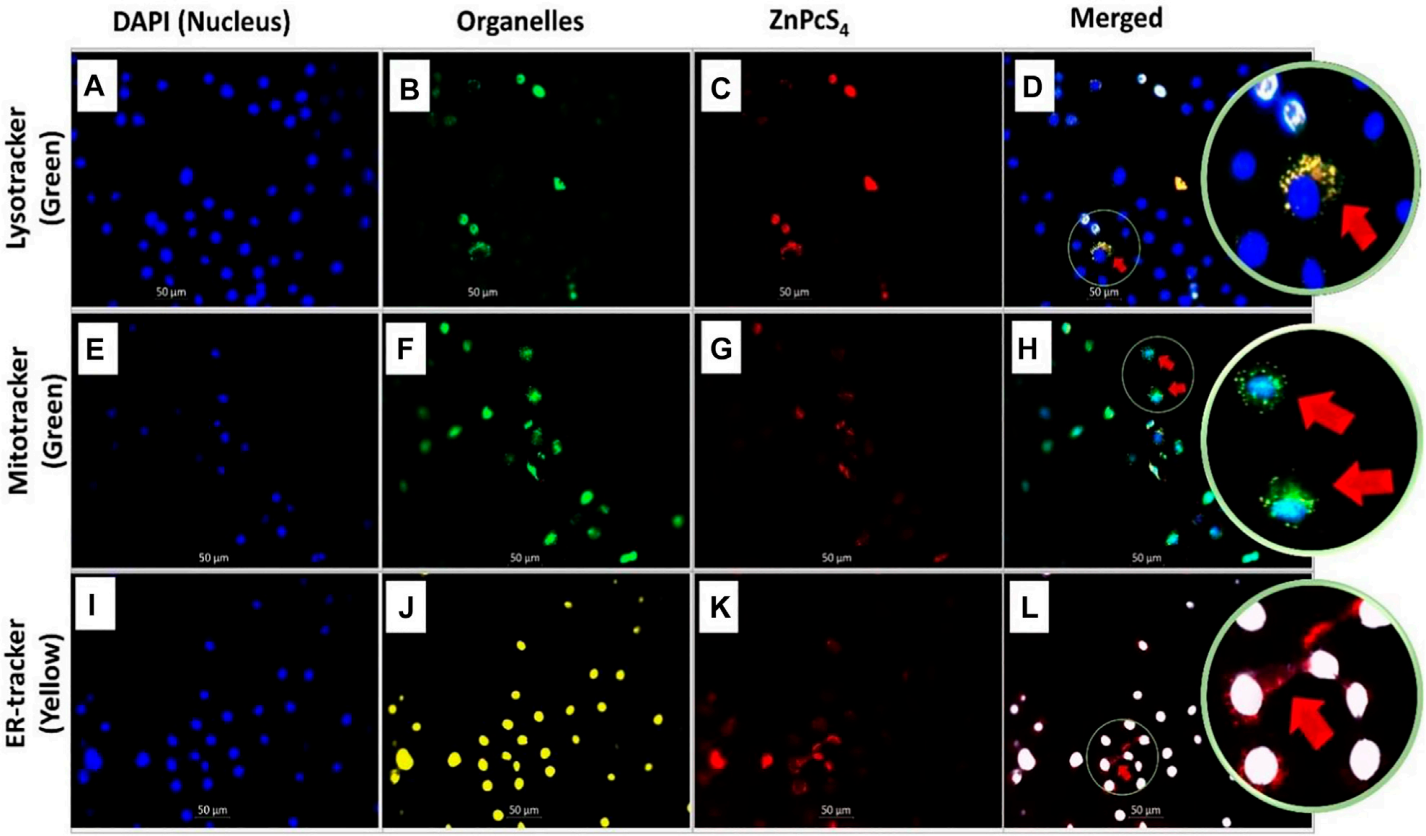
Subcellular localization of ZnPcS_4_ in MCF-7 breast cancer cells. DAPI blue fluorescence **(A,E,I)**, Lysotracker green fluorescence **(B)**, Mitotracker green fluorescence **(F)**, Endoplasmic reticulum (ER) Tracker yellow fluorescence **(J)**, ZnPcS_4_ red fluorescence **(C,G,K)**, and merged images **(D,H,L)**. (×200 magnification).

Fluorescence microscopy was used to visualize the intracellular localization of ZnPcS_4_ in MCF-7 breast cancer cells that were co-stained with DAPI (blue florescence) and different organelle specific fluorescent tracker dyes (Figure 3). However, red fluorescence (Figures 3C,G,K) indicates PS localization in different organelles of ZnPcS_4_ treated cells. In merged images, red fluorescence of ZnPcS_4_ in cells coincided with the green fluorescence of Mitotracker and Lysotracker dye, as illustrated in Figures 3D,H. This suggests that the PS localized in the mitochondria and lysosomes. The cells that were treated with ZnPcS_4_, stained with ER tracker dye (yellow florescence) and co-stained with DAPI showed no overlapping of the PS in the endoplasmic reticulum (Figure 3L). This indicates that the PS does not localize in the ER of MCF-7 breast cancer cells.

### 3.3 Dose Response Studies

#### 3.3.1 Morphological Analysis

**FIGURE 4:**
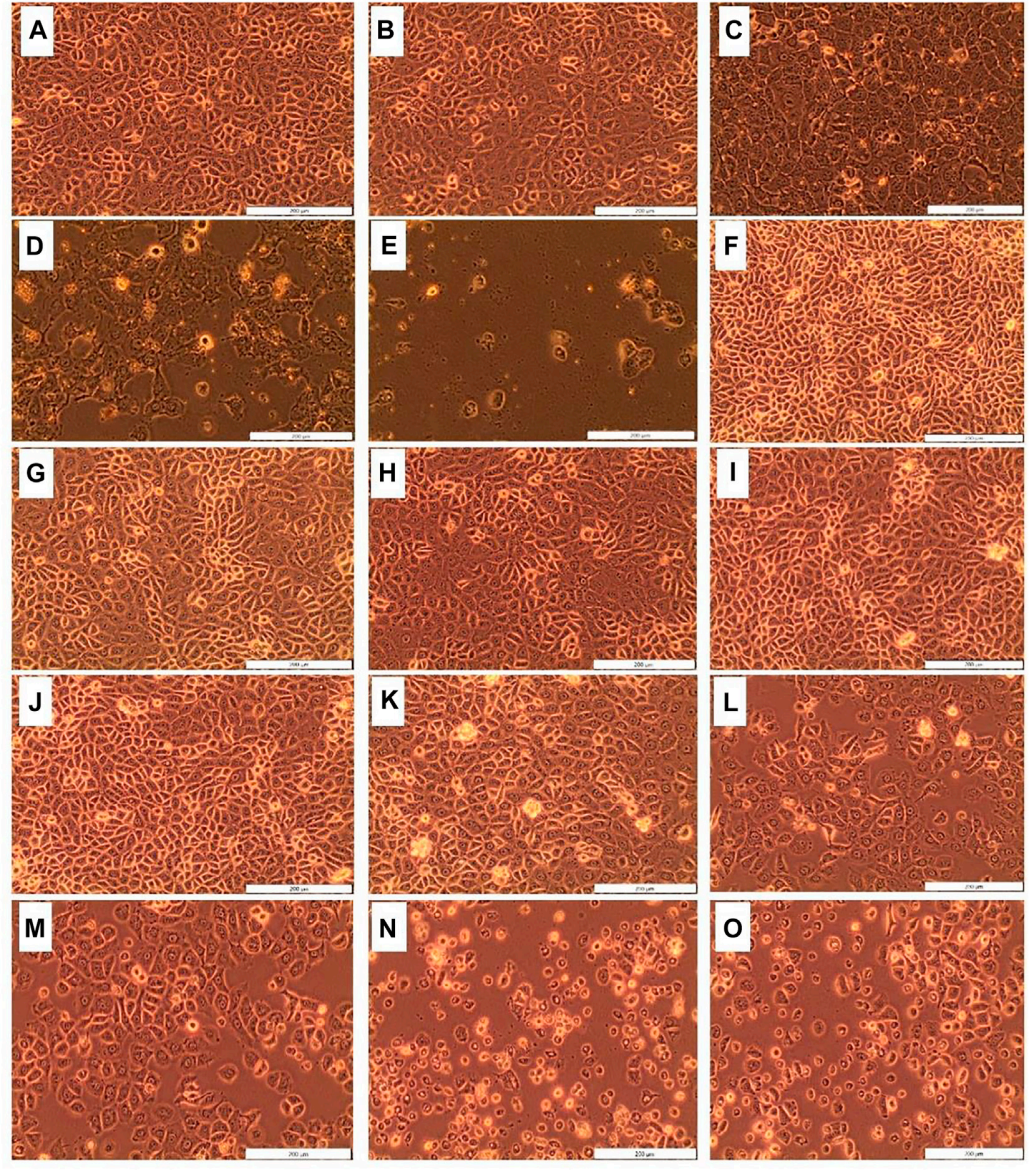
Cellular Morphology of MCF-7 cells post-treatment with *D. anomala* methanol root extract and ZnPcS_4._ Untreated cells **(A)**; cells + laser **(B)**; *D. anomala* 25 μg/ml **(C)**; *D. anomala* 50 μg/ml **(D)**; *D. anomala* 100 μg/ml **(E)**; ZnPcS_4_ 5 μM **(F)**; ZnPcS_4_ 10 μM **(G)**; ZnPcS_4_ 20 μM **(H)**; ZnPcS_4_ 40 μM **(I)**; ZnPcS_4_ 60 μM **(J)**; ZnPcS_4_ 5 μM + laser **(K)**; ZnPcS_4_ 10 μM + laser **(L)**; ZnPcS_4_ 20 μM + laser **(M)**; ZnPcS_4_ 40 μM + laser **(N)**; and ZnPcS_4_ 60 μM + laser **(O)**.

The morphological changes (i.e., blebbing, shrinkage, and vacuolization) in MCF-7 breast cancer cells treated with *D. anomala* methanol root extract and ZnPcS_4_ with and without irradiation is shown in Figure 4. No significant morphological changes were displayed in untreated cells 1), and irradiated cells 2) using a 680 nm diode laser with a consistent fluency of 10 J/cm^2^. Similar morphological pattern was observed in cells treated with various doses of ZnPcS_4_ (5, 10, 20, 40, and 60 μM) (Figures 4F–J). Dose-dependent morphological changes were observed in cells treated with various doses of *D. anomala* methanol root extract (25, 50, and 100 μg/ml) (Figures 4C–E). In a similar manner, ZnPcS_4_ treated, and irradiated cells displayed significant morphological changes (Figures 4K–O) when compared to untreated cells (Figure 4A).

#### 3.3.2 Trypan Blue Dye Exclusion Assay (Cell Viability)

**FIGURE 5:**
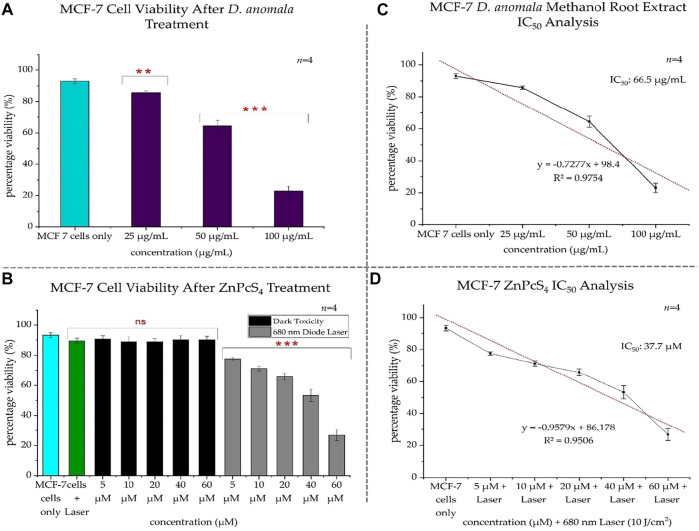
MCF-7 trypan blue exclusion cell viability after treatment with *D. anomala* methanol root extract and ZnPcS_4_ **(A,B)**. *D. anomala* IC_50_ **(C)** and ZnPcS_4_ mediated PDT IC_50_ **(D)**. The figures depicted in the graphs are mean ± standard errors of *n* = 4. No significance (ns), **p < 0.01 and ***p < 0.001 indicate significant differences between the mean values of the control and experimental groups.

The viability of the control cells in the first experimental model was 93%, whereas that of *D. anomala* treated cells were 85.75, 65, and 23% post-treatment with 25, 50, and 100 μg/ml respectively (Figure 5A). Based on the results obtained from trypan blue dye exclusion assay, the IC_50_ concentration was determined by plotting a linear regression perfect fit. Using the linear equation (y = −0.7277x + 98.4), the IC_50_ concentration was found to be 66.5 μg/ml (Figure 5C). In the second experimental model, 93.25% of control cells were viable. Whereas the cells treated with laser light showed no significant decrease in viability (89.5%) when compared to the control cells suggesting that irradiation using a 680 nm diode laser at 10 J/cm^2^ has no phototoxic effects on MCF-7 cells. Cells that were treated with ZnPcS_4_ but not irradiated showed no dark toxicity effects while ZnPcS_4_ treated cells that were irradiated showed a dose-dependent decrease in cell viability when compared to the control cells (Figure 5B). Subsequently, a linear regression perfect fit (y = −0.9579x + 86.178) with a greater regression (R^2^ = 0.9506) was used to determine the IC_50_ (37.7 µM) for ZnPcS_4_ mediated PDT (Figure 5D).

### 3.4 Combination Therapy of *D. anomala* and ZnPcS_4_ Mediated PDT

#### 3.4.1 Morphological and Viability Analysis

**FIGURE 6:**
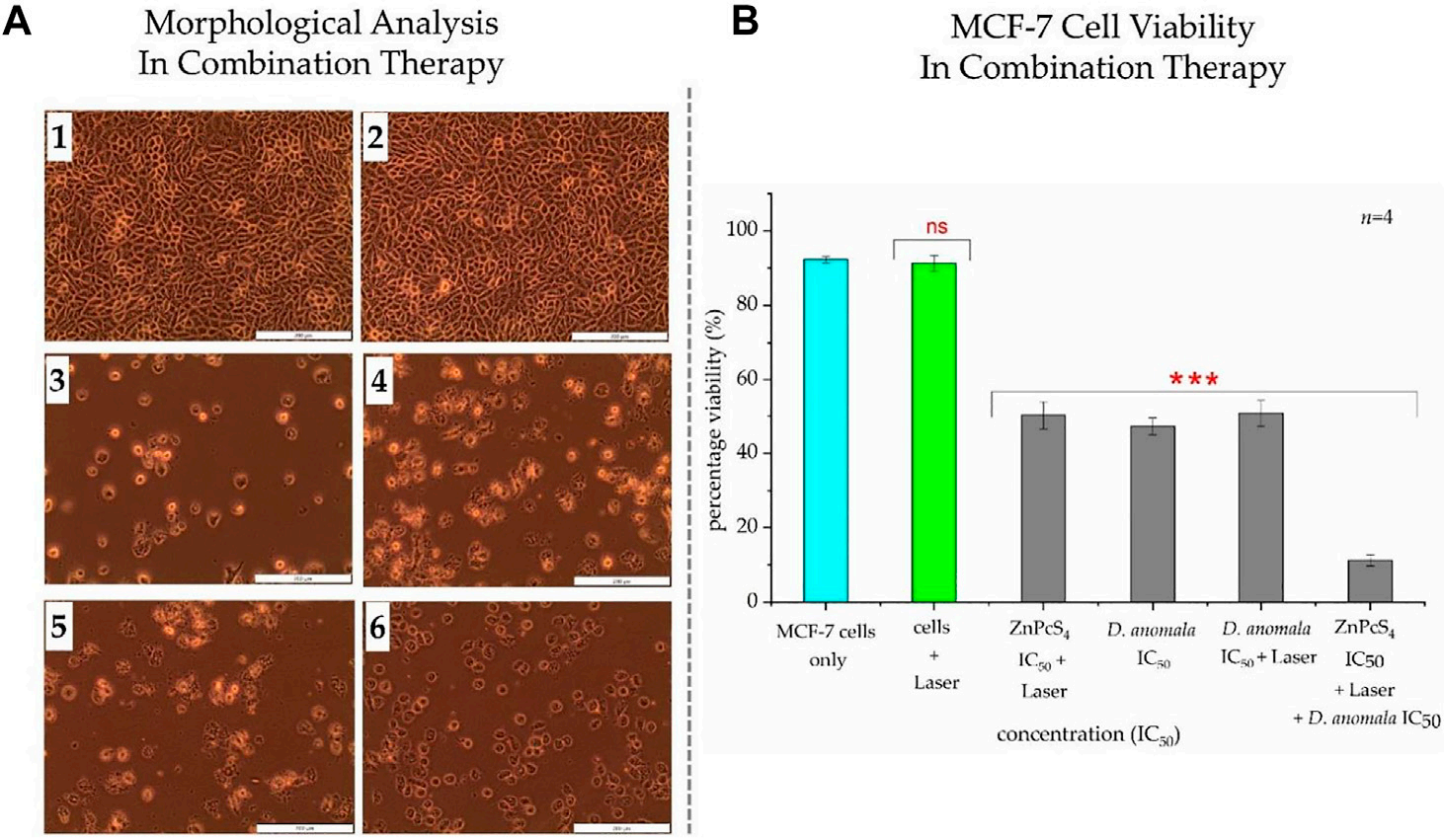
Cellular Morphology **(A)** and trypan blue exclusion cell viability **(B)** of MCF-7 cells post-treatment with *D. anomala* IC_50_ and ZnPcS_4_ IC_50_. Untreated cells (1); cells + 680 nm laser (10 J/cm^2^) (2); ZnPcS_4_ IC_50_ + 680 nm laser (10 J/cm^2^) (3); *D. anomala* IC_50_ (4); *D. anomala* IC_50_ + 680 nm laser (10 J/cm^2^) (5); ZnPcS_4_ IC_50_ + 680 nm laser (10 J/cm^2^) + *D. anomala* IC_50_ (6). The figures depicted in the graph **(B)** are mean ± standard errors of *n* = 4 with significant decrease in viability *p* < 0.001*** in all cells treated with IC_50_’s. Morphology (×200 magnification).

Morphological analysis in combination therapy displayed no changes in untreated and laser only treated cells (Figures 6A1,2). Significant morphological changes were observed in cells treated with ZnPcS_4_ IC_50_ (Figure 6A3), *D. anomala* IC_50_ (Figure 6A4) and *D. anomala* IC_50_ irradiated using a 680 nm diode laser (10 J/cm^2^) (Figure 6A5). Similarly, the combination of the two IC_50_’s (Figure 6A6) displayed substantial changes in morphology such as blebbing, loss of membrane integrity, and shrinkage when compared to control cells which maintained their normal morphology (Figure 6A1). Trypan blue dye exclusion assay was performed on both untreated and treated cells. No significant decrease in viability was observed in cells treated with laser alone. A significant decrease in cell viability was observed across all treatment groups of *D. anomala* IC_50_ and ZnPcS_4_ IC_50_. In a similar manner, a strong significant decrease in cell viability was observed in combination experiments (Figure 6B).

#### 3.4.2 Flow Cytometry (Annexin V-FITC/PI)

**FIGURE 7:**
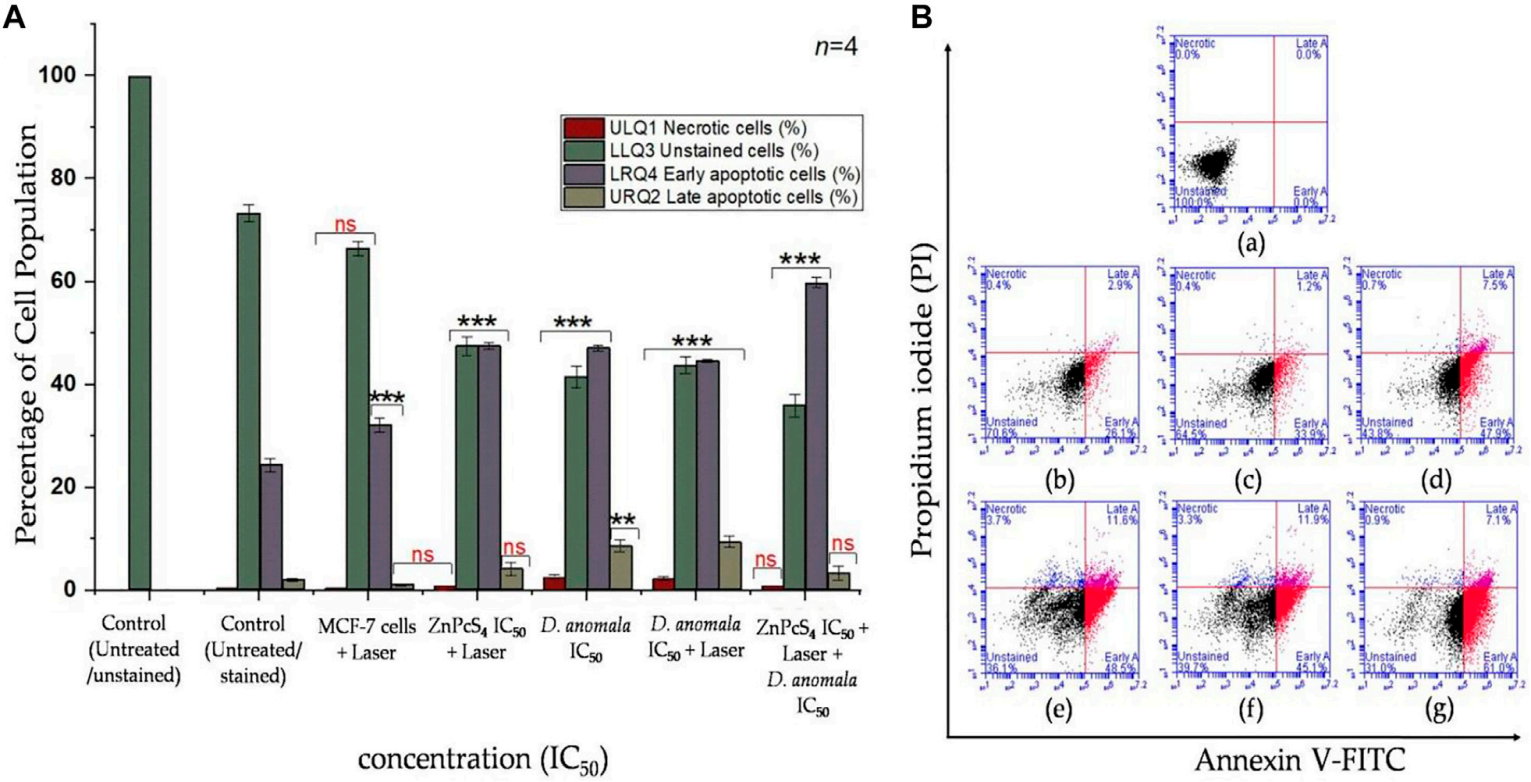
Annexin V-FITC/PI flow cytometry results **(A)** and its best scatter grams **(B)** in combination therapy. Untreated unstained (a), untreated stained (b), cells + 680 nm diode laser (10 J/cm^2^) (c), cells + ZnPcS_4_ + 680 nm diode laser (10 J/cm^2^) (d), cells + *D. anomala* IC_50_ (e), cells + *D. anomala* IC_50_ + 680 nm diode laser (10 J/cm^2^) (f), and cells + ZnPcS_4_ + 680 nm diode laser (10 J/cm^2^) + *D. anomala* IC_50_ (g). The figures depicted in the graphs are mean ± standard errors of *n* = 4. No significance (ns), **p < 0.01 and ***p < 0.001 indicate significant differences between the mean values of the control and experimental groups.

When compared to untreated and unstained MCF-7 cells, cells treated with a laser showed a significant increase in early apoptotic cell population (*p* < 0.001). The major cause of rise in early apoptosis can be linked to the normal programmed cell death. While there was no significant increase or decrease in necrotic, live unstained, and late apoptotic cell population observed. Compared to untreated/live and stained cells, cells treated with IC_50_ concentration of ZnPcS_4_ and received irradiation showed a significant decrease in unstained cell population and a significant increase in early apoptotic cell population, with no significant increase in necrotic and late apoptotic cell population. The cells treated with IC_50_ concentration of *D. anomala* alone revealed a significant drop in unstained/live cells (*p* < 0.001) and a significant rise in necrotic (*p* < 0.001), early (*p* < 0.001), and late apoptotic (*p* < 0.01) cell population. The cells that were treated with *D. anomala* and irradiated revealed a significant drop in unstained cells (*p* < 0.001) and a significant rise (*p* < 0.001) in necrotic, early, and late apoptotic cells (Figure 7A). Further, the combination of the two IC_50_’s of *D. anomala* and ZnPcS_4_ displayed a strong significant drop in unstained/live, and a significant increase in early apoptotic cell populations (*p* < 0.001). In addition, no significant increase or decrease in necrotic and late apoptotic quadrants was observed when compared to untreated/live stained MCF-7 cells (Figure 7A). Figure 7B illustrates the best schematic depiction of the scatter grams acquired.

#### 3.4.3 Immunofluorescence (Bax, p53, and Caspase 3 Expression)

**FIGURE 8:**
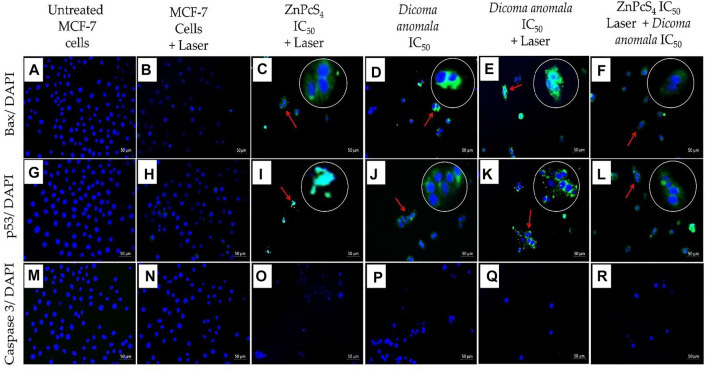
Expression of Bax, p53, and caspase 3 in MCF-7 cells post-treatment with D. anomala and ZnPcS_4_. DAPI (blue fluorescence) and apoptotic proteins FITC (green fluorescence). No expression of Bax **(A,B)**, no expression of p53 **(G,H)**, and no expression of caspase 3 **(M–R)**. Increased expression of Bax **(C–F)**, and p53 protein **(I–L)**. (x200 magnification).

The expression of apoptotic proteins (Bax, p53, and caspase 3) in treated cells were qualitatively analyzed by immunofluorescence. Two filter channels; DAPI (blue fluorescence for the nucleus) and FITC (green fluorescence for apoptotic protein expression) were used. With reference to Figure 8, Bax, p53, and caspase 3 expressions were evaluated in cells treated with individual as well as in combination of *D. anomala* and ZnPcS_4_ shows an increased expression of Bax when compared to untreated and laser treated cells which did not show any Bax expression (Figures 8A,B). Likewise, the expression of p53 in MCF-7 was significantly increased in experimental group’s i, j, k, and l (Figure 8). On the other hand, 680 nm diode laser (10 J/cm^2^) treated cells showed no p53 expression of (Figure 8H). Interestingly, no expression of caspase 3 was observed in any experimental groups (Figures 8M–R).

### 3.5 Discussion

Currently, PDT has been viewed as a promising therapeutic modality for different forms of cancer. When compared to conventional treatments, PDT offers relatively higher specificity and targeting with minimal side effects (Senapathy et al., 2020). To effectively eradicate cancer and avoid recurrence, most cancer patients may require one or more therapy. The reason to why most patients may be subjected to one or more therapies is because some of the common treatment modalities employed in the treatment of cancer are less effective when administered in monotherapy (Rutten et al., 2015; Tohme et al., 2017). PDT may be used as a supportive treatment for primary therapies (Wang et al., 2020). In recent years, plant-derived bioactive compounds use in the treatment of different alignments has increased and many medicinal plants are being evaluated for the presence of potential phytochemicals with therapeutic effects (George et al., 2016). In addition, plant derived PSs with low laser irradiation properties could offer more therapeutic effects in the field of cancer research than commercially manufactured PSs, which are associated with some high degree of toxicity (Isaac-Lam and Hammonds, 2019). In the present study, we explored phytochemical profiling and therapeutic efficacy of *D. anomala* methanol root extract in monotherapy as well as in combination with ZnPcS_4_ mediated PDT in MCF-7 breast cancer cells.

Soxhlet extraction is the most commonly used method for the extraction of phytocompounds (Zhang et al., 2018). Chloroform, ethanol, and methanol are the most often utilized solvents in phytochemical extractions (Kasiramar, 2018). A study by Naser et al. (2018) demonstrated anticancer effects of caffeoylquinic acids on gastric adenocarcinoma (AGS) cell line. These caffeoylquinic acidic compounds induced the cell cycle arrest in the G1 phase and eventually apoptosis. Murad et al. (2015) reported anticancer effects of 5-caffeoylquinic acid on human colon adenocarcinoma (HT-29) cell line. They revealed that 5-caffeoylquinic acid inhibited HT-29 cell proliferation and arrested cell cycle in the G0/G1 phase. It has also been reported that caffeoylquinic acids inhibit tumor cell progression and induces tumor cell death through the mitochondrial mediated death pathways (Skała et al., 2018). Similar to caffeoylquinic acids, dicaffeoylquinic acids were also reported to possess anticancer effects on human colon cancer (HCT-116) cell line (Gouthamchandra et al., 2017; Zhou et al., 2020). Similar to these reports our study showed the presence of two active secondary metabolites i.e., caffeoylquinic and dicaffeoylquinic acidic compounds in *D. anomala* methanol root extract (Figures 2B,C) and the anticancer effects of *D. anomala* reported in this study could be due to the presence of one or the combination of the two compounds.

The subcellular localization of PS is critical in PDT since it impact on overall PDT efficacy (Sekhejane et al., 2014). Physico-chemical properties of PSs, such as charge, lipophilicity, partition coefficients, and three-dimensional structure influence their uptake, subcellular localization, and aggregation within cells, which is further influenced by cellular transport mechanisms and the organelle’s membrane potential (Bayona et al., 2017). Tynga et al. (2013) and Manoto et al. (2012) reported that ZnPcS mix largely localizes in the mitochondria and lysosomes of MCF-7 breast cancer cells. Similar to these studies, our study evaluated the localization of ZnPcS_4_ in MCF-7 breast cancer cells by using specific organelle markers (i.e., DAPI, endoplasmic reticulum, mitochondrial, and lysosomal trackers). It was found that ZnPcS_4_ preferentially localized in the mitochondria, lysosomes, and the cytoplasmic matrix (Figures 3D,H).

Cancer cells display various characteristics during the cell death. One such example is morphological changes such as membrane blebbing, rounding, and shrinkage (Kumar et al., 2015; Khajah and Luqmani, 2016). Tripathy et al. (2020) reported that silver nanoparticles synthesised from *D. anomala* Sond. Root extract revealed apoptotic features such as nuclear condensation and tumor cell shrinkage, demonstrating its anticancer effects on MCF-7 breast cancer cells. Manoto et al. (2012) also revealed the similar morphological features in A549 lung cancer cells post-treatment with ZnPcS mix mediated PDT. In a similar manner, the present study also revealed that *D. anomala* and ZnPcS_4_ mediated PDT elicited substantial morphological changes in MCF-7 cells when administered in monotherapy as well as in combination therapy. However, no morphological changes were observed in MCF-7 cells received only irradiation. This clearly suggests that 680 nm diode laser at 10 J/cm^2^ does not have any thermal effects on cancer cell killing (Figure 4B and Figure 6A).

The present study revealed a dose dependent decrease in cell viability after treatment with *D. anomala* methanol root extract and ZnPcS_4_ mediated PDT when administered in monotherapy and in combination therapy. This clearly indicates the enhanced cytotoxic effects of *D. anomala* methanol root extract in combination with ZnPcS_4_ mediated PDT. Similar studies have reported that plant extracts can enhance the efficacy of phthalocyanine mediated PDT of cancer (George et al., 2016; Senapathy et al., 2020). A remarkable progress has been achieved in drug design of apoptosis-modulating cancer therapies in the recent decade, and numerous human clinical studies are now underway (Sharma et al., 2021). Most importantly, unlike necrosis, apoptosis does not initiate the activation of inflammatory activities, thus making it a preferred cell death mechanism for cancer therapies than necrosis (Hu et al., 2021). In addition, understanding the mechanisms of cell death induced by various plant-derived phytocompounds is very important in drug development. Although the cell death mechanisms induced by *D. anomala* root extracts are not well established. A comprehensive review by Muniraj et al. (2019) propose apoptosis of being one of the common cell death mechanism induced by numerous plant–derived bioactive compounds. The present study revealed necrotic and apoptotic cell death mechanisms induced by *D*. *anomala* extract and PS. However, apoptosis was the most predominant cell death mechanism in all combination therapy experimental groups. Our results are also in line with the reports of Naidoo et al. (2019), who showed apoptosis is the most prominent cell death mechanism in A375 metastatic melanoma cells post 24 h treatment with ZnPcS_4_ and gold nanoparticles (AuNPs) in PDT.

The apoptotic cell death mechanism can be initiated by various stimuli (e.g., physiological and pathological stimuli), it is worth noting that not all cells undergo the same pathway of cell death (Elmore, 2007). Two major apoptotic pathways (intrinsic and extrinsic apoptotic pathways) have reported to play a crucial role in the regulation of tumor cell population (Kashyap et al., 2021). The extrinsic apoptotic pathway is mainly initiated by conditions of extracellular environment that interact and trigger death ligand to induce apoptosis (Fulda and Debatin, 2006). Through the intrinsic signaling apoptotic pathway, induction of apoptosis can be initiated by various cytotoxic stimuli and pro-apoptotic signal-transducing proteins (e.g., Bax and Bak) that target the mitochondria and induce mitochondrial outer membrane permeabilization (MOMP) (Green and Llambi, 2015). In certain cancer cell lines, the use of PDT and many other plant-derived bioactive compounds have been reported to induce apoptosis via the activation and interactions of anti-apoptotic proteins (e.g., Blc-2, Bcl-w, and Mcl-1), pro-apoptotic proteins (Bax and Bak), p53, and effector caspases (Mitsunaga et al., 2007; Iqbal et al., 2017; Banjara et al., 2020).

Bax is a member of Bcl-2 family of proteins and is one of the most common pro-apoptotic proteins. When up regulated the Bax proteins induce the activation of mitochondrial caspase-mediated apoptotic pathway (Kim and Kim, 2018). Briefly, the role of activated Bax in tumor cell death is to induce MOMP which in turn results in the release of mitochondrial pro-apoptotic factor cytochrome c into the cytoplasmic matrix. In the cytoplasmic matrix, cytochrome c is reported for recruiting apoptotic protease activating factor-1 (Apaf-1) which when bound together forms an apoptosome (Mizuta et al., 2007). Many studies have revealed that the formation of apoptosome within a cell results in the activation of the caspase cascade: the effecter of apoptotic activities (Bao and Shi, 2007; Brentnall et al., 2013). It has also reported that MCF-7 breast cancer cells do not express effecter caspase 3 (Jänicke, 2009). The p53 tumor suppressor protein is another essential apoptotic protein that is upregulated when a cell is under stress (Pflaum et al., 2014). A number of studies have reported p53 of regulating the cell cycle, and inducing cell death via the intrinsic apoptotic pathway (Beckerman and Prives, 2010; Pflaum et al., 2014). Like these reports, our study displayed a strong expression of Bax, and p53 in all treatment groups. However, no expression of caspase 3 was observed in all treatment groups of the combination therapy. Previous studies have also revealed similar results in relation to the expression of Bax, and p53 proteins (Weilbacher et al., 2014).

**FIGURE 9:**
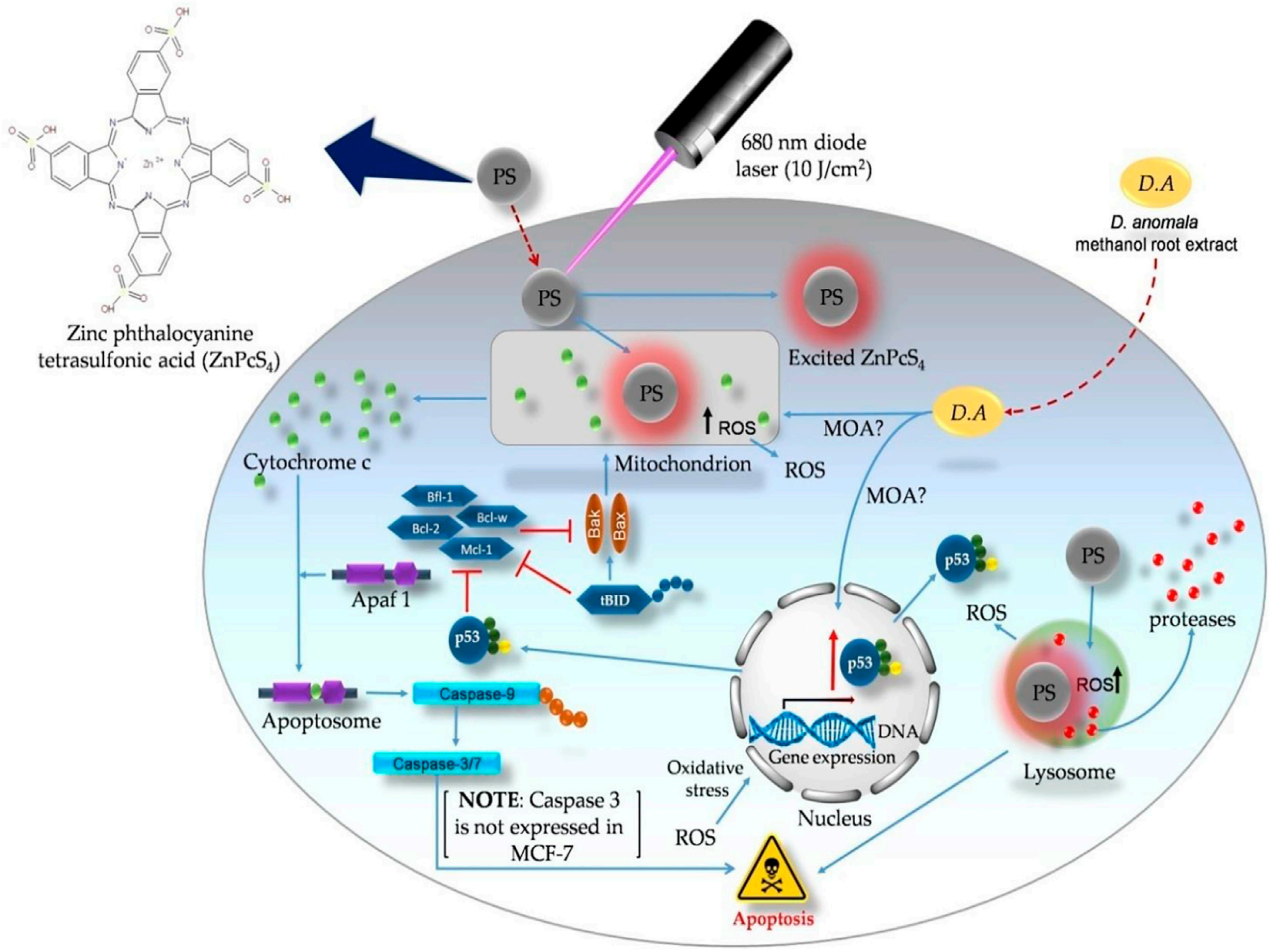
Probable apoptotic signaling pathways induced by *D. anomala* methanol root extract and ZnPcS_4_ mediated PDT in MCF-7 breast cancer cells.

Figure 9 depicts the representation of probable cell death signaling pathways induced by *D. anomala* methanol root extract and ZnPcS_4_ mediated PDT in MCF-7 cells based on the results from this study. With reference to flow cytometry and immunofluorescence results, there are two probable apoptotic signalling pathways (mitochondrial, and lysosomal-mediated degradative apoptotic pathways) initiated by *D. anomala* and ZnPcS_4_ in individual as well as in combination therapy. The proposed mitochondrial-mediated probable intrinsic apoptotic pathway is initiated post-treatment with *D. anomala* and ZnPcS_4_. In this pathway, the stimuli from the two treatments resulted in the recruitment of pro-apoptotic protein Bax. With reference to many literature, the activation of Bax by the BH3 interacting-domain death agonist (tBID) leads to Bax homo-oligomerization, MOMP, and indirect initiation of the caspase cascade (Malkesman et al., 2012; Wang and Tjandra, 2013). Several studies and evidence from our study, we therefore suggest that the localization of the PS in the lysosomes induced oxidative stress on the lysosomes which eventually results in lysosomal membrane permeabilization (LMP) and the release of degradative lysosomal enzymes (proteases) into the cytoplasmic matrix of treated cells. The release of proteases into the cytoplasmic matrix has been reported of causing substantial damage on cellular components (Kavčič et al., 2017).

## 4 Conclusion

This study revealed the anticancer effects of *D. anomala* and ZnPcS_4_ mediated PDT in MCF-7 cells when administered in monotherapy and enhanced the cytotoxic effects with decreased viability of cells in combination therapy. The findings from this study shows that combining the therapies increase the sensitivity of cancer cells to the treatments. Combination therapies can further reduce dose dependence by lowering the dosage of commercially synthesized photosensitizers, which could in turn limit the side effects of PDT. It was also found that apoptosis was the most prominent form of cell death in different experimental groups. We also explored the expression of apoptotic proteins post-treatment, and the results revealed the expression of Bax, p53 and no expression of caspase 3. The outcomes from this study clearly demonstrate the identification of two major secondary metabolites (caffeoylquinic and dicaffeoylquinic acid) that may be the reason for the reported anticancer effects.

## Data Availability

The original contributions presented in the study are included in the article/Supplementary Material, further inquiries can be directed to the corresponding author.
